# Their Place in the Hospital Budget

**Published:** 1914-04-25

**Authors:** 


### THE RIGHT TREATMENT OF LEGACIES.

### Their Place in the Hospital Budget.

During the ten years—1903-1912—the hospitals of London received an annual average of £289,800 from legacies. As a very large proportion of this sum was left free from any restriction as to allocation to endowment or other purposes, and as experience shows that the average annual receipts from legacies vary little over long periods, the hospitals concerned may, subject to the possibility of a great social upheaval, count on at least a quarter of a million from this source for some years to come.

It is the practice of most hospitals to spend all free income (which includes unrestricted legacies) on current expenditure. The instances where it has been found possible to save out of surplus income are few and far between. We acknowledge that it is a fantastic thought, but we should like to consider for a space what would happen to the poor needing hospital treatment if suddenly the hospitals should find themselves without the income derived from legacies. Yet it was seriously urged at the annual meeting of the London Homœopathic Hospital that it is right to invest legacies and use only the interest for current expenditure. This view was supported by the Chairman of the Kent County Ophthalmic Hospital in a letter to the *Times*, in which the writer went so far as to say there was an "implied trust," when a testator left money to a hospital, under which the legacy should be treated as capital. The Chairman of the London Hospital joined in the correspondence and took the opposite view that current cash is intended to be spent on current needs, and that it would be unfair to relieve posterity of its responsibilities.

The wisdom and the morality of spending legacies has been argued across the board-room table times without number; and the spending party, backed by the financial exigencies of our institutions, have so far won the day. Conservative finance requires that a voluntary hospital must secure that it shall have free money invested as a minimum reserve to an amount equal to five times its average yearly ordinary expenditure. When such a reserve has been secured a voluntary hospital is certainly at liberty to act on the view expressed by the Chairman of the London Hospital, which has been steadily and wisely increasing its reserves through sound management, in late years especially. No hospital committee can be financially safe which does not act continuously upon this principle. Then from the point of view of the ethical defence of the spending policy it may be said that testators should be alive to what they are doing when they make their wills, and presumably would, when leaving money, give instructions if they wish it treated in any way out of the ordinary course. That some, at least, are quite aware and willing that their benefactions shall be spent on ordinary outgoings is known to most hospital executives, in whose memory there must be many cases where legators have made doubly sure by indicating that the money shall be used for ordinary expenditure.

As Lord Knutsford says: "Why should a gift I make now, to take effect on my death, be treated differently to a gift which takes place in my life? Any hospital can strike an average of its legacies just as easily as it can of its donations, which are an equally uncertain quantity." It is, however, more to the point to deal with the practical side of the question at the present moment. An influential secretary to an important London hospital propounds the following position: Let us suppose that the "investment" party is in the right and able to convince the other side, and that the whole of the London hospitals, in their desire to do that which is just, refrain, all at once, from availing themselves of the use of legacies for current needs. The work would have to be cut down immediately to fit in with altered circumstances, for it would be obviously childish to invest and then sell out or borrow against the investments. There are 10,152 hospital beds in London, and the average income from legacies (calculated over ten years) is £289,000. After we have deducted £89,000 for legacies with a trust attached, and for sums which at present for one reason or another are invested, there remain £200,000. We will apply the reduction in the volume of work to beds, as most of the money spent is upon in-patients. Taking it that an in-patient costs £75 a year we should have to close 3,000 beds (roughly, 30 per cent. of the whole). In this calculation an allowance has been made for the well-known fact that, when beds are closed to save money, certain large standing charges still remain, and consequently extra beds have to be dispensed with to balance such outgoings."

He continues: "We have admitted that the idea is fantastic, but the lesson from it is perhaps worth considering and should enhance our appreciation of the voluntary system. Could public assistance in other forms in London possibly cope with such a burden suddenly thrown upon its shoulders and adequately provide for the accommodation of 3,000 persons, not in chronic physical condition but suffering from urgent illnesses or accident? Even if the Metropolis alone took the step the situation would be almost sufficient to cause politicians to turn from other large matters of State and temporarily apply themselves exclusively to this problem. Nor would the shortage be only for a little time. At the end of five years, making all allowance for accrued investments, 2,520 beds would still be closed, at the end of ten years 1,920, and not until a quarter of a century had elapsed would the hospitals regain their balance, to find, perhaps, that their laborious savings, at the cost of great suffering on the part of the poor, might be swept by the large hand of a Chancellor of the Exchequer into a common pool to maintain some vast national health scheme.''

The incidence of legacies is always interesting to students of hospital work. The percentage of legacies left by subscribers and donors may be reckoned to average under 10 per cent. This seems to show that there is little in the theory that it is a testator's wish to provide an annual gift after death. If this were so, surely he would have shown some practical proof of his belief in the value of annual support during lifetime!

Hospital authorities have never satisfactorily been able to satisfy themselves how legators become interested. To what stray seed, borne by the wind of publicity, a legacy is due few can tell. It may be through the advice of a solicitor or a family physician; or an appeal or an advertisement in some publication may have more than earned its cost after many years. Our experience and a mass of evidence in our experience points conclusively to the fact that since King Edward actively interested himself in the voluntary hospitals and studied carefully each year the facts given in Burdett's "Hospitals and Charities," that book has been used by wealthy people who leave legacies and by solicitors, much as "The Official Intelligence of the Stock Exchange is used by financiers when they wish to select a hospital or charity for their gifts and legacies. So Burdett's "Hospitals and Charities" and the agony and hospital columns in the *Times* bring undoubtedly regular and increasing funds to those voluntary hospitals who employ these agencies regularly and wisely. These facts are not sufficiently realised and much money is wasted in useless advertisements in consequence. That some of the lists one sees published from time to time in the newspapers have been carelessly put together one cannot doubt. Within the last few years legacies have been left to institutions maintained by rates, and much perplexity has arisen as to how to use the funds so acquired. Again, very frequently we see left to various institutions, in the same will amounts quite out of proportion to the requirements of the respective objects so benefited.

Students of the matter are aware that, given a fair amount of publicity, a hospital sooner or later enters a legacy zone, and when it is in that happy position a regular volume of assistance (averaging the receipts over a moderate number of years) may be relied upon from the source of legacies. Looking over the records of two well-known London hospitals which have by courtesy been made available for the purpose, we find some interesting particulars. In each case the hospital was founded about the middle of last century. One of these institutions began to receive legacies two years after foundation, and in about twelve years the number of legacies falling due began to average ten each year, and has kept at about that rate ever since. In the other instance no legacies were received for twenty years, and the steady average did not commence until twelve years after the receipt of the first legacy. The average here, again, has remained at about the same figure ever since.

It is difficult to draw a lesson from these statistics. The first advent of legacies may have depended upon the amount of publicity that the hospital was able to obtain during its early life; but as publicity should be a cumulative thing, one might have expected the averages steadily to increase from the beginning in each case. We have, of course, been dealing with dates when legacies were received, and not with dates when wills were made. In the first of our examples we find that the average interval between the date that the will was made and the date when it was proved was three years and four months; the longest interval was twenty years and four months, and the shortest thirteen days. In the other example the average was just over four years; the longest period that the hospital had to wait was twenty-two years and two months, and the shortest one month and six days.

Each of these hospitals has had lean and fat years, but never has the yield been below the average for more than five years running. Perhaps experience elsewhere may be the same, that, having reached the legacy zone, a hospital in drawing up its budget may confidently base calculations upon a steady income from legacies.

